# Quantification of Biocatalytic Transformations by Single Microbial Cells Enabled by Tailored Integration of Droplet Microfluidics and Mass Spectrometry

**DOI:** 10.1002/anie.202204098

**Published:** 2022-05-31

**Authors:** Konstantin Wink, Marie van der Loh, Nora Hartner, Matthias Polack, Christian Dusny, Andreas Schmid, Detlev Belder

**Affiliations:** ^1^ University of Leipzig Institute of Analytical Chemistry 04107 Leipzig Germany; ^2^ Department Solar Materials Helmholtz Centre for Environmental Research (UFZ) 04318 Leipzig Germany

**Keywords:** Droplet Microfluidics, Mass Spectrometry, Single Cells, Whole-Cell Catalysis, Microreactors

## Abstract

Improving the performance of chemical transformations catalysed by microbial biocatalysts requires a deep understanding of cellular processes. While the cellular heterogeneity of cellular characteristics, such as the concentration of high abundant cellular content, is well studied, little is known about the reactivity of individual cells and its impact on the chemical identity, quantity, and purity of excreted products. Biocatalytic transformations were monitored chemically specific and quantifiable at the single‐cell level by integrating droplet microfluidics, cell imaging, and mass spectrometry. Product formation rates for individual *Saccharomyces cerevisiae* cells were obtained by i) incubating nanolitre‐sized droplets for product accumulation in microfluidic devices, ii) an imaging setup to determine the number of cells in the droplets, and iii) electrospray ionisation mass spectrometry for reading the chemical contents of individual droplets. These findings now enable the study of whole‐cell biocatalysis at single‐cell resolution.

## Graphical abstract

A microfluidic platform for investigating the biocatalytic conversion of substrates into products at the single‐cell level by mass spectrometry is presented. The platform was used to obtain product formation rates for individual *Saccharomyces cerevisiae* cells.

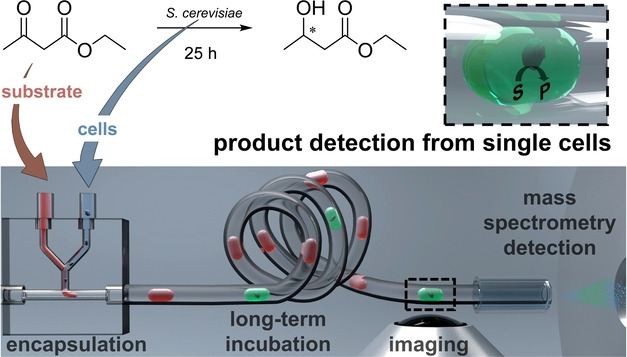

## Introduction

Our current knowledge on the performance of whole‐cell biocatalysts is based on the averaged performance of millions of cells, all acting as individual, catalytically active units. Recent studies revealed significant cell‐to‐cell heterogeneity using transciptomics,^[1]^ metabolomics,^[2]^ and proteomics^[3]^ analyses. However, the race is still on to study whole‐cell catalysed chemical conversions at the single‐cell level. Comprehension of the causes of cellular catalytic heterogeneities would significantly contribute to our understanding of how cells work and how biocatalysts and processes could be optimised for an intended chemical conversion. For biotransformations towards target molecules, detailed chemical information is required, including turnover, substrate and product scope, as well as stereo‐ and regioselectivity of the conversion. The only analytical technique to answer these questions at a single‐cell level is arguably mass spectrometry (MS). The field of single‐cell MS is vast and multifaceted in terms of instrumentation and also concerning the object of interest. Organisms investigated range from giant nerve cells or mammalian cells to tiny bacteria and yeast cells.^[4]^ So far, single‐cell‐MS reports were almost exclusively concerned with examining the cell content in mammalian cells.^[5]^ This is understandable as the concentration of the most commonly studied metabolites is high and their amount scales with cell volume. If the concentrations are significantly above the detection limit of the analytical method, the challenge can be narrowed down to achieving a sufficiently high spatial resolution to probe just one cell in an ensemble. This is possible, for example, with high spatial resolution imaging MS, which can achieve resolutions in the micrometre range.^[6]^

If the cells are isolated prior to the actual analysis, this can also be achieved with other MS‐techniques such as matrix‐assisted laser desorption/ionisation‐MS, as shown for single‐cell metabolomic studies on yeast by the Zenobi group.^[7]^ These works are currently the state‐of‐the‐art in single‐cell mass spectrometry of small cell types and have contributed to an idea of their metabolic cell heterogeneity and the chemical composition of the cell interior. However, from the perspective of whole‐cell biocatalysis, it would be intriguing to know how the surrounding cell solution changes as a result of the cellular catalytic process. One of the grand challenges in analysing biocatalytic conversions is the simple question of how much substrate is consumed and how much product is formed per unit of time by one single cell, along with the chemical specificity and purity of the product.

However, significant analytical challenges arise when chemical conversions at a single‐cell level are investigated. Due to dilution effects during product accumulation in the surrounding solution, the expected product concentrations are minuscule and gradually build up over the reaction time. It is thus not surprising that corresponding single‐cell studies are scarce and restricted to large, but from an organic catalysis point of view, less interesting organisms such as mammalian cells^[8]^ or other large cell types.^[9]^ The field of microbial catalysis at the single‐cell level is still unexplored, but recent studies approached this absolute limit. We have investigated different routes for achieving single‐cell resolution using microreactors in integrated chip devices. In a continuous flow method, we captured microbes in a dielectrophoretic cage and collected the secreted product in a capillary for off‐line analysis by nano‐electrospray ionisation (nESI)‐Fourier transform ion cyclotron resonance MS.^[10]^ Although using state‐of‐the‐art MS technology, this allowed product quantification only down to a number of 19 cells at low throughput (one sample per day). Droplet microfluidics in combination with ESI‐MS has been useful for probing minuscule reactor volumes ‐ at high throughput.^[11]^ With this approach, we monitored earlier the biosynthesis of lysine from a lower limit of 10 cells of *C. glutamicum* via droplet/ESI‐MS hyphenation.^[12]^ However, this did not allow us to reach the single‐cell level, mainly because the ESI‐MS product signals were below the detection limit due to ion suppression by the surfactant. The detergent was necessary to avoid coalescence of the droplets in the compact chips during long‐term incubation to achieve significant product levels.

We here present a microfluidic platform that overcomes previous limitations and enables the quantification of biocatalytic products at the single‐cell level. Our microfluidic approach demonstrates how single‐cell specific reactivities of a biocatalytic chemical transformation can be accessed via droplet/MS coupling. Using glass chips for droplet generation and fluorinated capillaries to store separate surfactant‐free droplets, we quantified product titers in distinct droplets by combining this capillary approach with online ESI‐MS analysis. Unambiguous correlations between the product concentration and the number of cells in the droplets were achieved by in‐line droplet imaging inside the transparent capillaries. Combining these technologies enabled the determination of cell‐specific product formation rates for an enantioselective reduction of a keto ester to the corresponding hydroxy ester, a non‐natural compound produced by Sa*ccharomyces cerevisiae*, and the quantification of biocatalytic heterogeneity between individual catalysts.

## Results and Discussion

**Figure 1:**
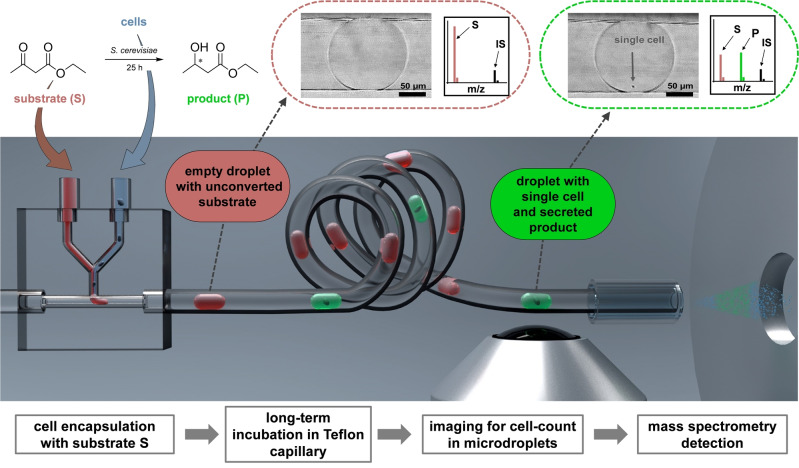
Schematic representation of the presented capillary‐based approach to study single‐cell catalysed conversions via electrospray‐ionisation mass spectrometry detection. The platform includes a glass chip for cell encapsulation, a droplet storage and incubation in a Teflon capillary, and a droplet analysis (cell imaging as well as MS detection). A substrate (S) and a cell suspension are mixed and encapsulated into droplets that are seamlessly transferred into a Teflon capillary for extended reaction times. In droplets without cells, the substrate is not converted (red dotted circle including a photography of an empty droplet, and a schematic mass spectrum). In droplets with single cells, the substrate is reduced to the corresponding hydroxy product (P) by *S. cerevisiae* (green dotted circle including a photography of a droplet with one cell, and a schematic mass spectrum*)*. The accumulated product is quantified with the use of an internal standard (IS) in distinct droplets by mass spectrometry after determining the cell count in each droplet prior to the detection.

Figure 1 visualises our approach for monitoring whole‐cell biocatalysis at the level of single microbial cells. The herein developed integrated analytical microfluidic platform enabled the encapsulation of cells with defined amounts of a substrate (S) into nanolitre droplets, the incubation of cell‐loaded droplets to accumulate the catalytic product (P), and droplet analysis by cell imaging and subsequent MS analysis. We investigated the catalytic activity of individual *S. cerevisiae* cells. As a model reaction, we studied the whole‐cell catalysed reduction of the keto ester ethyl‐3‐oxobutanoate^[13]^ to ethyl‐3‐hydroxybutyrate, a widely used precursor in fine chemical syntheses.^[14]^ For microfluidic operations, e.g., mixing or cell encapsulation, we applied monolithic fused‐silica glass chips that were microstructured via selective laser‐induced etching.^[15]^ This enabled a dead volume‐free connection of standard capillaries with an outer diameter (o.d.) of 360 μm and allowed the generation of uniform droplets of constant volumes in the nL range. In this way, the cell suspension and the reactant solution were mixed and segmented into droplets by a vertically introduced fluorinated continuous phase (perfluorodecalin). The generated droplets were seamlessly transported to a fluorinated capillary for long‐term incubation. The catalysis products thus accumulated in high enough concentrations for quantification via online droplet/ESI‐MS. For imaging and MS analysis of distinct droplets, the string of droplets was transported directly to the ionisation source of the mass spectrometer. A common issue in single‐cell studies using droplet microfluidics is that most droplets are empty due to statistical cell encapsulation from a dilute suspension, following the Poisson distribution. For assigning the cell number within a droplet to the MS signal, the droplet trace was video‐recorded with a mobile microscope^[16]^ in front of the mass spectrometer. Time‐stamping allowed the assignment of the droplets to distinct MS peaks, enabling to correlate MS signal with the cell numbers inside the droplets. The droplets′ contents were then ionized via a commercially available capillary electrospray coaxial sprayer unit.^[17]^ Applying a standard ESI‐MS setup facilitates a seamless transfer to other laboratories while guaranteeing robust droplet MS coupling.^[18]^ Using fluoropolymer capillaries allowed us to store well‐spaced 3‐nL‐sized droplets without surfactants that could interfere with analyte ionisation, as is often encountered in droplet/ESI‐MS analysis.[^12^, ^19^]

The eukaryotic unicellular yeast *S. cerevisiae* was selected as a model microbial biocatalyst due to its broad application and long‐standing history as a host strain in biologically catalysed organic syntheses from lab to industrial scale. In addition to its extensive substrate acceptance in numerous reaction environments, it can catalyse various chemical reactions, such as C−C bond formation and cleavage, reductions, or oxidations.^[20]^

Initial studies were done in shake flasks to monitor and evaluate the reaction performance of the cells at the millilitre scale (results are summarized in the Supporting Information in Figure S1). The reaction medium used was a solution of volatile ammonium acetate, which is MS‐compatible^[21]^ and well suited for biocatalytic conversions.^[12]^

**Figure 2:**
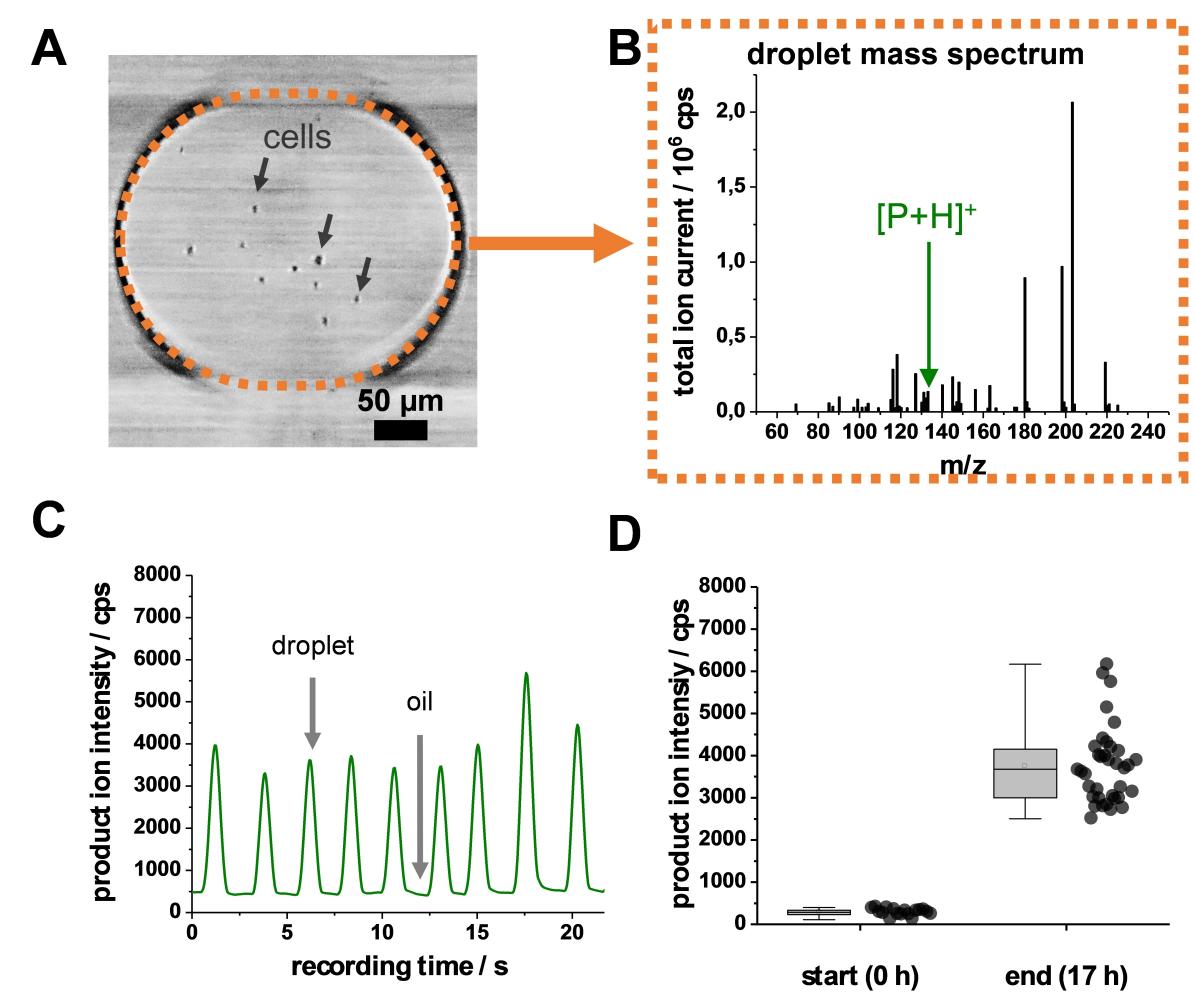
Evaluation of the investigated model reaction in 15 nL droplets. A) Microscopic image of a droplet containing cells (*S. cerevisiae*) in a 300 μm i.d. and 1.59 mm o.d. tubing. B) Typical mass spectrum of a distinct droplet with the signal corresponding to the protonated product ion [P+H]^+^. C) Typical MS ion count trace of the product ion for a reaction with 10–15 cells per droplet (visible as a peak) in a continuous oil phase (visible as a signal valley). D) Evaluation of droplets at the start and the end (17 h) of a reaction. Each data point represents the product ion intensity stemming from an individual droplet with 10–15 cells per droplet (N=53).

As a starting point for microscale analysis, we monitored the reaction in 15 nL droplets at cell numbers of 10–15 cells per droplet to prove the feasibility of the experimental microfluidic setup. Droplets were generated in a tee cross with a bore diameter of 250 μm and stored in a polytetrafluoroethylene (PTFE) capillary with a 300 μm inner diameter (i.d.) and an o.d. of 1.58 mm (Figure S2, Supporting Information). Using a portable microscope enabled enumerating the individual droplets and counting the number of cells per droplet before entering the ESI‐sprayer. Figure 2A exemplifies this via a typical light microscopic droplet image obtained. Yeast cells could be identified as dark objects that displayed a characteristic ellipsoidal shape with the occurrence of cell buds.

For MS analysis of 15‐nL‐sized droplets, droplets were transferred to a smaller capillary (75 μm i.d. and 360 μm o.d.) which led directly to the ESI‐sprayer in front of the mass spectrometer. Figure S3a/b (Supporting Information) illustrates an example of seamless droplet transition enabling the droplet/MS analysis. For the electrospray ionization process, a coaxial sheath‐liquid assisted ESI sprayer was used, in which droplets emerging out of the incubation capillary to the sprayer tip flowed into a coaxial sheath fluid and were nebulized by a nebulizing gas toward the MS inlet (Figure S3C). Generally, each droplet generates a corresponding mass spectrum (Figure 2B), allowing a non‐target analysis of reaction products. To achieve maximum sensitivity, the product signal was detected in multiple reaction monitoring mode with a precursor/product ion transition at the mass‐to‐charge ratio (*m*/*z*) pair 133.1/87.1.

A typical product ion intensity trace is shown in Figure 2C. Each peak represents the detection of a droplet, while the arrival of the oil phase causes a signal valley. Droplets showed an increase in the product ion intensity after a reaction time of 17 h, indicating product formation. Figure 2D shows this for droplets in boxplot form for a 17 h reaction demonstrating the product detection in 15 nL droplets in capillary‐based systems via ESI‐MS.

**Figure 3:**
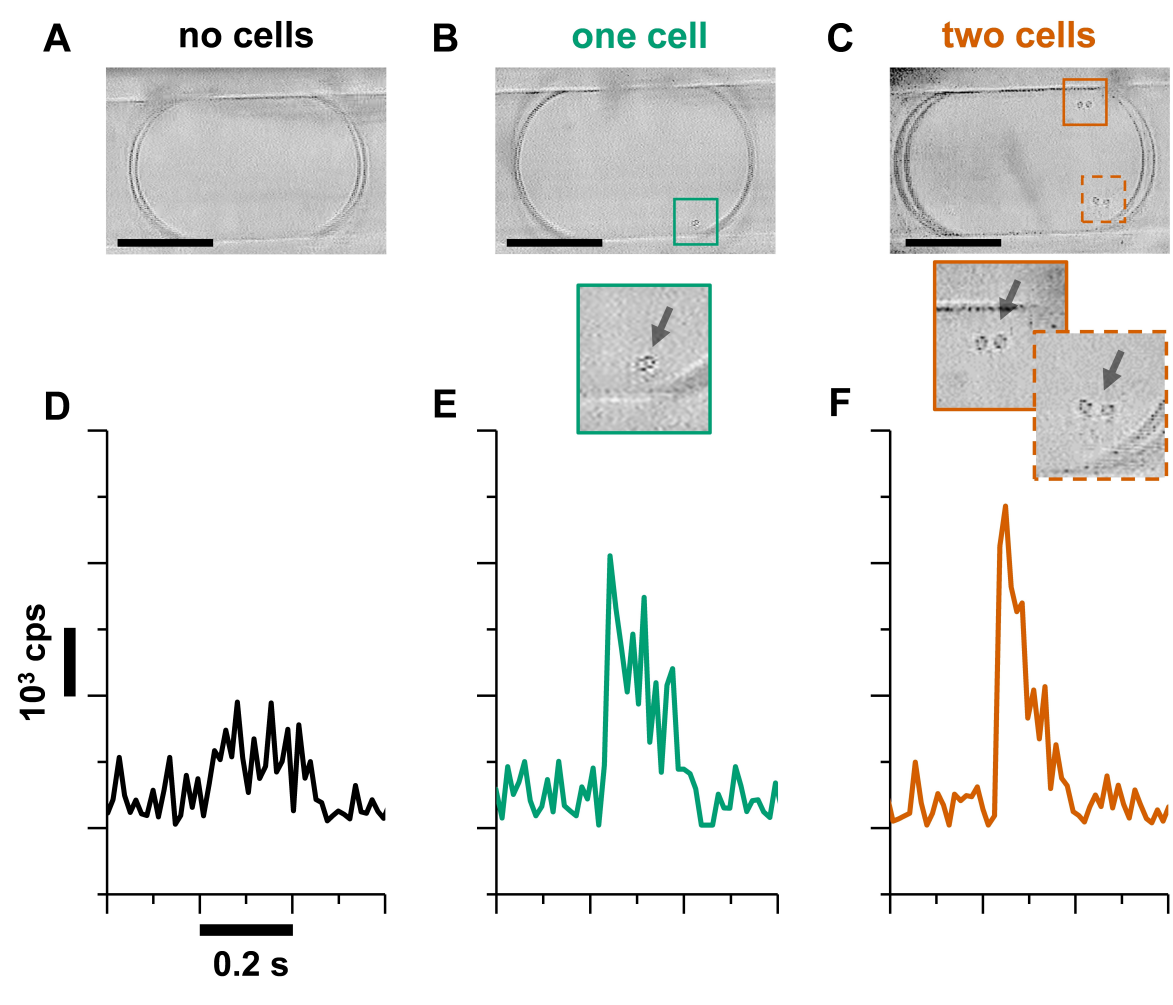
Visual (A)–(C) and ESI‐MS‐based (D)–(F) droplet analysis at single‐cell resolution. Comparison of empty droplets containing the reaction mixture without cells (A), and droplets containing one cell (B) and two cells (C) at the end of a reaction (25 h) with corresponding product ion traces for droplets without cells (D), and droplets containing one cell (E) and two cells (F). A typical bud formation can be seen in the cells (*S. cerevisiae*). Scale bar: 100 μm.

After the successful evaluation of our analytical platform, we aimed toward smaller cell numbers inside droplets. To advance to the single‐cell level, the reactor volume was further reduced to increase the product concentration inside the droplets. By using microfluidic glass chips with a tee‐junction bore diameter down to 130 μm, and a 150 μm i.d. and 360 μm o.d. perfluoroalkoxyalkane (PFA) capillary for storage, droplets in the low single‐digit nL volume range could be generated (Supporting Information, Figure S4). Droplets of 3 nL volume were generated and stored in a PFA capillary leading directly to the ESI sprayer. Before droplet MS analysis, the droplet trace was imaged by video microscopy to correlate cell numbers in the droplet with the corresponding MS ion traces. Respective images are shown in Figure S5, and relevant videos with droplets including zero (Video S1) or one cell (Video S2) are provided in the Supporting Information. The distribution of cells per droplet matched with the theoretical Poisson distribution^[22]^ (Figure S6A, Supporting Information), e.g., ≈82 % of droplets were empty, ≈16 % contained one cell per droplet, while ≈2 % contained more than one cell per droplet. The evaluated droplet library of empty droplets and droplets containing one or more cells was correlated to the corresponding MS data. Empty droplets exhibited a product ion intensity that correlated well with the signal level obtained for droplets containing only the unconverted reaction matrix (Figure S6B, Supporting Information). Figure 3 shows exemplary traces of the product ion signal for empty droplets and droplets with one or two cells. Yeasts were identified in the droplets with a typical bud formation protruding from the yeast cell. Although different droplets may show similar MS signal intensities, the optical analysis allowed the assignment of the number of cells to specific MS peaks. An exemplary overview of additional product ion traces is shown in Figure S7 to demonstrate the variability of the droplet ESI‐MS detection (Supporting Information).

**Figure 4:**
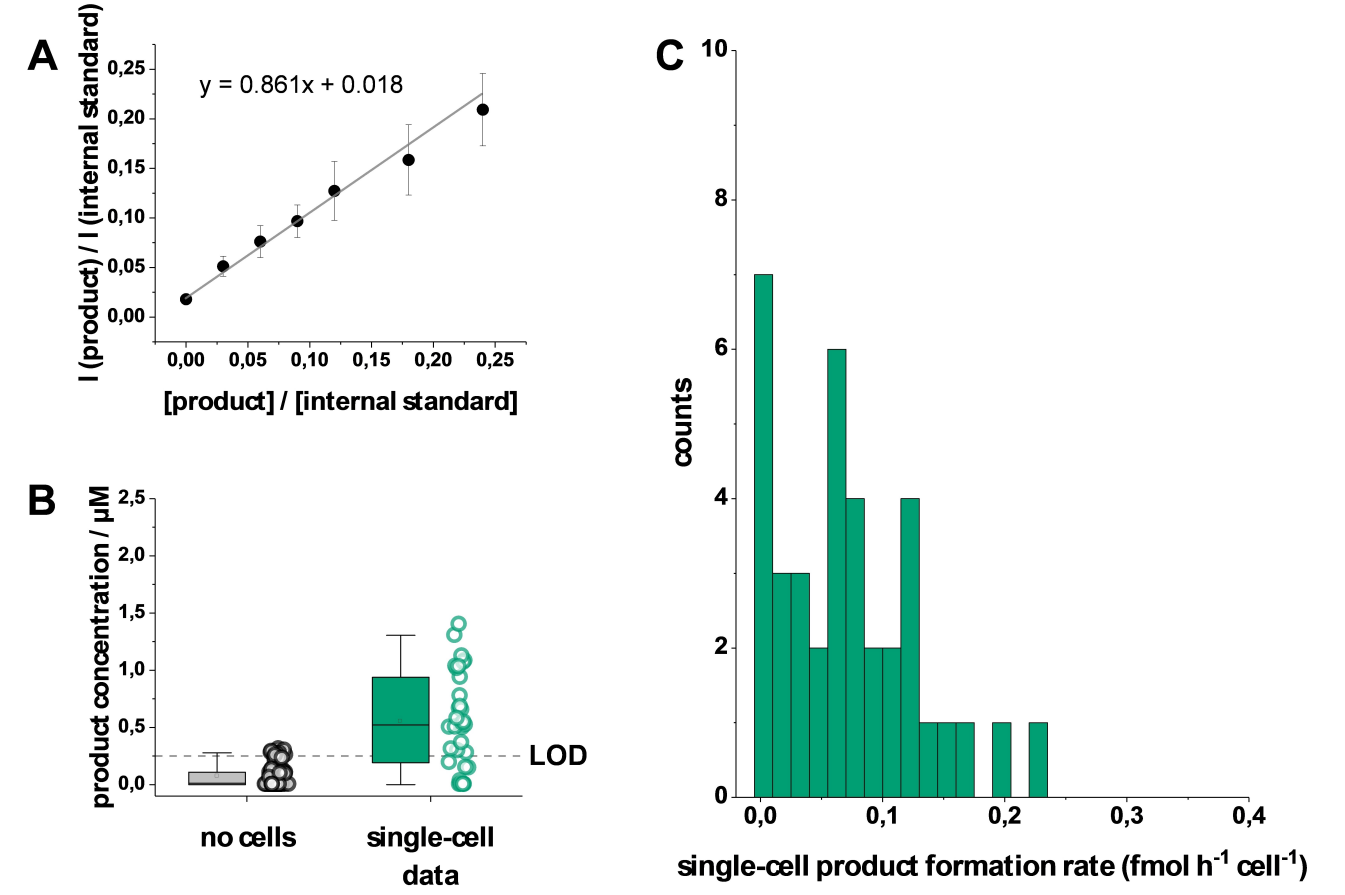
Determination of single‐cell specific product formation rates. A) Calibration curve of droplets with the product and an internal chlorinated standard. B) Boxplot of the concentration per droplet after 25 h reaction time for empty droplets (no cell) and droplets containing one cell (single‐cell data); N=129. A dotted line indicates the LOD (0.25 μM). C) Single‐cell product formation rates for individual cells are plotted as a histogram; N=38.

A chlorinated derivative of the product was used as an internal standard to quantify the product concentration in individual droplets. Figure 4A shows the corresponding calibration curve of droplets containing different product concentrations and 10 μM of the internal standard. The limit of detection (LOD) was calculated as 0.25 μM (3.3 SD/m, SD: standard deviation at x_0_, m: slope). Theoretical considerations confirmed the product concentration observed for droplets containing single cells. Corresponding quantitative single‐cell data are shown in Figure 4B. The product was quantified by product‐ion peak identification, background correction of the peak intensity (determined prior to experiments), and quantification with the corresponding internal standard‐ion peak intensity. For no‐cell‐containing droplets, product concentrations ranged from zero up to the LOD. For droplets containing a single cell, concentrations were up to approximately 1.5 μM, well above the limit of quantification (3×LOD). The mean product concentration in droplets with single cells was calculated as 0.55 μM. At this concentration, the relative standard deviation of the corresponding signal intensity was 21 % (calculated from the calibration curve in Figure 4 A, i.e., the product in the reaction matrix). This provided to differentiate the performance of single cells in the range of 0.55±0.19 μM. Since it is not possible to derive the cell number exclusively from the MS signal due to the cell‐to‐cell heterogeneity, the differentiation of the cell number was ensured by the additional optical method. This combination allowed the assignment of product concentrations to the respective cell number, i.e., for droplets with more than one cell, as shown for two and three cells per droplet in Figure S8 (Supporting Information).

This method allowed us to determine the product formation rate per cell (fmol h^−1^ cell^−1^) at the single‐cell level. Figure 4C shows the evaluation of the reaction with droplets containing only single cells. The single‐cell data of the catalytic activity revealed significant cell‐to‐cell heterogeneity between the isogenic cells, with product formation rates ranging up to 0.23 fmol h^−1^ cell^−1^ for high‐producing cells. 26 % of the investigated cells had product formation rates below 0.017 fmol h^−1^ cell^−1^. The absolute mean value was 0.071 fmol h^−1^ cell^−1^. Although single‐cell product formation rates from the reaction studied are not available in the literature, population‐based estimates^[23]^ of approximately 0.03–0.2 fmol h^−1^ cell^−1^ are in a similar range to our obtained data. Overall, these findings demonstrate the feasibility of capturing reactivities of single microbial cells for the conversion of xenobiotic compounds. While we used an ESI‐MS compatible reaction medium with low salt content, it is generally possible to adapt the reaction medium to more complex media with higher salt loading, as recently^[24]^ demonstrated for the monitoring of the catalytic activity of a free enzyme with droplet/ESI‐MS in 100 mM sodium phosphate buffer. Compared to approaches employing continuous flow reactors^[10]^ or stationary droplet arrays^[25]^ with MS detection, we have demonstrated a substantial increase in throughput with a parallelised segmented flow reactor approach while achieving single‐cell resolution. The sampling rate was approximately 0.8 samples/s, which is in the range of common droplet/MS platforms.^[26]^ We anticipate that our approach, in combination with advanced automated droplet imaging^[27]^ will significantly improve throughput along with progress in MS data acquisition. The combination with novel droplet manipulation technologies such as mass‐activated droplet splitting/sorting^[28]^ and on‐demand droplet collection^[29]^ could facilitate promising downstream processes like the isolation and cultivation of high‐producing cells from droplets. Future efforts will focus on improving the ionisation of droplets to minimise the dilution caused by the sheath‐liquid‐assisted sprayer used.

## Conclusion

With the technology presented, it is now possible to investigate the biocatalytic conversions at the single‐cell level by mass spectrometry. Our framework constitutes a novel tool enabling understanding and optimising whole‐cell biocatalytic systems. In this study, we investigated the product formation rates of asymmetric whole‐cell catalysis for the reduction of a xenobiotic keto ester to the corresponding hydroxy ester. Although MS cannot distinguish enantiomers as such, this approach gets us close to observing enantioselectivity at the single‐cell level.^[30]^ To achieve this goal, future studies will explore the in situ generation of diastereomeric adducts^[31]^ as well as the use of pseudoenatiomeric compounds^[32]^ to enable enantioselective mass spectrometric determination in single droplets. Given the recent advances in the analysis of isomers using ion mobility spectrometry (IMS),^[33]^ the combination of the technology presented here with droplet IMS^[34]^ is another avenue now paved toward enantioselective catalytic transformation studies at the single‐species level. Our results now allow detailed studies of the minimal catalytic unit, an isolated single cell, to better understand reaction heterogeneity, reaction mechanisms, metabolism, and biochemical network functions determining the selectivities and efficiencies of whole‐cell biocatalysts.

## Conflict of interest

The authors declare no conflict of interest.

1

## Supporting information

As a service to our authors and readers, this journal provides supporting information supplied by the authors. Such materials are peer reviewed and may be re‐organized for online delivery, but are not copy‐edited or typeset. Technical support issues arising from supporting information (other than missing files) should be addressed to the authors.

Supporting Information

Supporting Information

Supporting Information

## Data Availability

The data that support the findings of this study are available from the corresponding author upon reasonable request.
