# Supplementary material for: Quantification of Biocatalytic Transformations by Single Microbial Cells Enabled by Tailored Integration of Droplet Microfluidics and Mass Spectrometry
Source: Angew Chem Int Ed Engl. 2022 May 31;61(29):e202204098. doi: 10.1002/anie.202204098 (PMC9401594; doi:10.1002/anie.202204098)
Supplement: Supplementary file 1 — Supporting Information [file ANIE-61-0-s001.pdf]

Angewandte Chemie
Eine Zeitschrift der Gesellschaft Deutscher Chemiker

Supporting Information

# Quantification of Biocatalytic Transformations by Single Microbial Cells Enabled by Tailored Integration of Droplet Microfluidics and Mass Spectrometry

*K. Wink, M. van der Loh, N. Hartner, M. Polack, C. Dusny, A. Schmid, D. Belder**

# Supporting Information

## Table of content


| | | |
|---|---|---|
| Experimental Section | ... | S2 |
| Reduction of ethyl-3-oxobutanoate by *S. cerevisiae* in flask experiments | ... | S4 |
| Generation and storage of droplets | ... | S4 |
| Droplet transition between capillaries | ... | S5 |
| Monolithic tee junction chip | ... | S6 |
| Extracted still images of droplets with single cells | ... | S6 |
| Visual and ESI-MS-based droplet analysis at single-cell resolution | ... | S7 |
| Variability of droplet ESI-MS signals | ... | S7 |
| Correlation between droplets with different cell amounts to the corresponding product concentration per droplet | ... | S8 |
| Supplemental Videos | ... | S9 |
| References | ... | S9 |

## Experimental Section

### Droplet Generation

For the generation of 3 nL droplets, customised glass chips with a channel width and height of 130 µm were used. Off-the-shelf tee crosses with 250 µm bore diameter or 150 µm bore diameter could also be used for droplet generation, yet they showed a lack of uniformity during droplet generation. In the case of the tee cross with 250 µm bore diameter (stainless steel, ZT1C, Vici), 300 µm i.d. / 1.58 mm o.d. PTFE capillaries were used. For the glass chips and the tee cross with a 150 µm bore diameter (Polyether ether ketone, P-888 MicroTee, Idex), 150 µm i.d. / 360 µm o.d. PFA capillaries were used. The microstructured full-body fused silica chips were fabricated by a selective laser-induced etching (SLE) process (Fig. S4). The monolithic microstructures were introduced by a selective laser-induced etching process (FEMTOprint f200 aHead P2, Muzzano, Switzerland) followed by a wet etching step in hot potassium hydroxide (85°C, 8 mol/L). The microfluidic channels were inscribed with a 20x objective (LMH-20X-1064, N.A. = 0.40, Thorlabs, Bergkirchen, Germany) in a 100 mm x 1 mm fused silica wafer (Siegert Wafer GmbH, Aachen, Germany) with a pulsed laser radiation at 1030 nm with an energy of 230 nJ at 1000 kHz repetition rate and a pulse duration of 400 fs respectively. These approaches allowed the generation of 15 nL (250 µm bore) or 3 nL (130 µm - 150 µm bore) droplets. The flow rates were 5 µL/min for the discontinuous phase and 30 µL/min for the continuous phase for generating 15 nL droplets. To generate 3 nL droplets, the flow rates were 3 µL/min for the discontinuous phase and 15 µL/min for the continuous phase.

### Sample Preparation

Commercially available dry baker's yeast (*S. cerevisiae,* Dr. August Oetker Nahrungsmittel KG, Bielefeld, Germany*)* was used as the microbial catalyst. The cells were suspended in tap water with glucose as an energy source and shaken for 3 hours at 120 rounds per minute at room temperature in 10 mL flasks (KS-15, Edmund Bühler GmbH, Bodelshausen, Germany). Desired cell concentrations were obtained by measuring the optical density at 660 nm ($OD_{600}$) with a spectrophotometer (V-650 Jasco, Pfungstadt, Germany).[1] The cells ($OD_{600}$ ~ 1) were washed three times by centrifugation with the reaction medium (10 mM ammonium acetate, Merck KgaA, Darmstadt, Germany), including 0.6 mM glucose (Merck KgaA) and diluted to the desired cell concentration. Samples for the reaction were prepared by mixing a cell suspension with a cell concentration of 0.13 x $10^6$ cells/mL ($OD_{600}$ 0.01) with glucose and a solution containing the substrate ethyl-3-oxobutanoate (99%, Sigma-Aldrich GmbH (Taufkirchen, Germany), with both solutions containing 10 µM of the internal standard ethyl-4-chloro-3-hydroxybutyrate (96%, Sigma-Aldrich GmbH) in a tee cross before encapsulation. Thus, the dispersed phase consisted of the cell suspension (0.06 x $10^6$ cells/mL), 0.6 mM glucose, 20 µM substrate, and 10 µM internal standard in an ammonium acetate solution (10 mM). This solution was segmented into droplets by a vertically introduced continuous phase (perfluorodecalin, Fluorochem Ltd, Hadfield, U.K.) in a tee junction.

### ESI-MS Analysis of Droplets

After droplet formation and filling of the capillary with droplets, all flows were stopped and the inlet for the aqueous phase was unscrewed and blocked. The oil flow was reversed so that the nitrogen from the ionisation chamber was drawn back into the capillary over a length

of about 25 cm. The flow was stopped for droplet storage. Using the Teflon capillary (300 µm i.d. / 1.58 mm o.d.) for storing 15 nL droplets, a seamless connection to the sheath liquid-assisted ESI sprayer was made by inserting an emitter capillary (75 µm i.d. and 360 µm o.d.) into the storage capillary after widening (Supporting Information, Fig. S3). For the use of 3 nL droplets, the PFA capillary (150 µm i.d. / 360 µm o.d.) was used as the storage and emitter capillary, which was directly inserted into the sheath liquid-assisted ESI sprayer. For MS analysis, the oil flow rate was set to 0.7 µL/min for 3 nL droplets (for 15 nL droplets it was set to 10 µL/min) to deliver the droplets to the ionisation source. Using a portable microscope (custom-made by Thalheim Spezialoptik, Germany) with a 20x/0.5 UPlan FI objective (Olympus, Japan) and a white LED for trans-illumination, the droplet trace was recorded on video from the first droplet counted from the extracted nitrogen plug. The experiments were performed on an Agilent 6495C triple quadrupole instrument. The multiple reaction monitoring mode was used for MS analysis with specific precursor/product ion transitions for the analytes with corresponding mass-to-charge ratio pairs (m/z) of 131.1/85.1 for the substrate, 133.1/87.1 for the product, and 167.1/121.1 for the internal standard with a collision energy of 7 V and a dwell time of 1 ms for each transition. The sheath fluid ($H_2O$/MeOH, 50/50 vol%) flow rate was set to 15 µL/min for 15 nL droplets (20 µL/min for 3 nL droplets). The ionisation source parameters were 250 °C dry gas temperature, 15 L/min dry gas, 7 psi nebuliser pressure, and 3500 V MS inlet potential in positive mode. The video-recorded droplet trace was manually evaluated for a specific droplet classification (timestamp and cell number) and correlated with the corresponding MS ion traces. A product ion background noise was subtracted for each droplet, determined from artificial matrix mixtures without the product and yeast cells. For quantification, a chlorinated derivative of the product was used as an internal standard at a concentration of 10 µM.

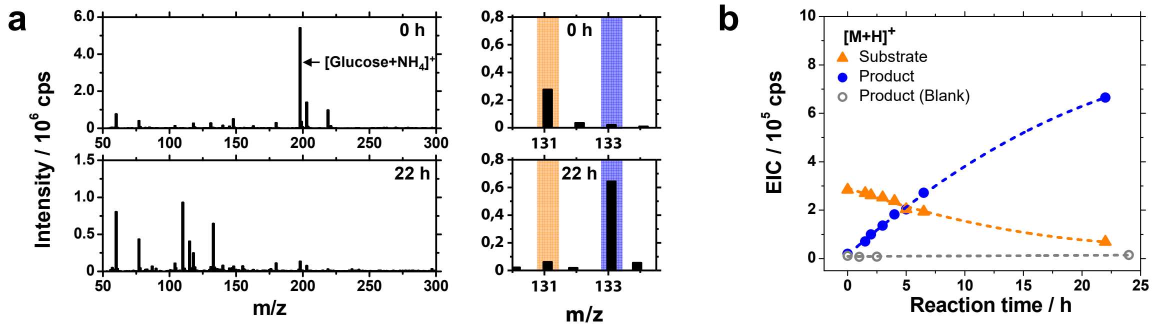


**Fig. S1** Reduction of ethyl-3-oxobutanoate by *S. cerevisiae* in flask experiments. (a) Mass spectra at the beginning (0 h) and end (22 h) of the reaction. (b) Reaction course of the extracted ion chromatograms $(M+H]^{+})$ for the substrate, the product, and the product signal of the blank. Reactions were carried out in shake flasks (reaction volume 10 mL) and analyzed by flow injection ESI-MS analysis (10 µL injection volume). The reaction medium was ammonium acetate solution (10 mM). The yeast cell concentration was 1.25 mg/mL, and the glucose concentration was 6.6 mM at the beginning. The concentration of the substrate was 100 µM at the beginning. The experiments were performed on an Agilent 6150B quadrupole mass spectrometer.

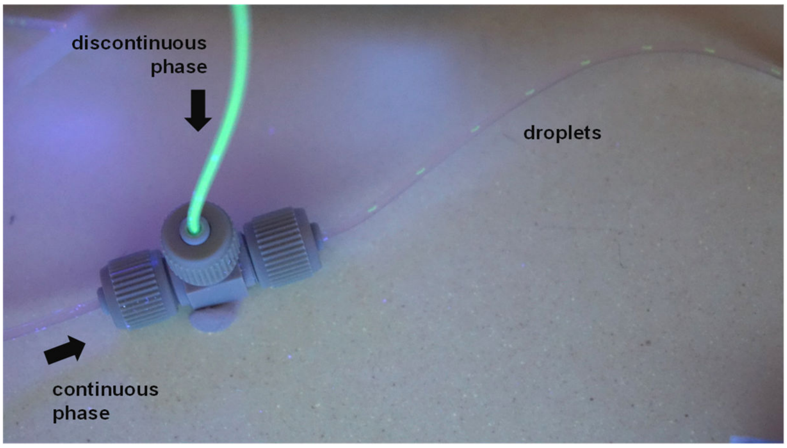


**Fig. S2** Generation and storage of droplets. (A) Droplet generation with a tee cross with a bore size of 250 µm and (B) droplet storage in a Teflon capillary (300 µm i.d. and 1.59 mm o.d). A fluorescent discontinuous phase was used (1 mM Fluorescein) and irradiated with UV light for visualization. Continuous phase: perfluorodecalin.

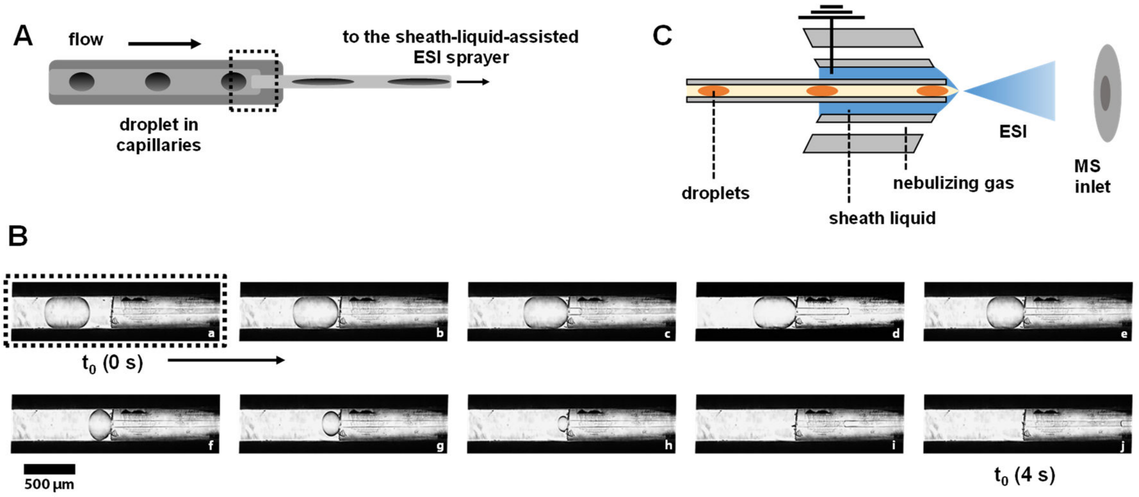


**Fig. S3** Droplet transition between capillaries and used ESI sprayer. (A) Schematic diagram of the transition. (B) Droplet transition from a storage capillary (300 µm i.d. and 1.59 mm o.d., PTFE) to an emitter capillary (75 µm i.d. and 360 µm o.d, PFA) for ESI. For the connection, the emitter capillary was inserted into the storage capillary after the entrance of the storage capillary was widened with a fused silica capillary (360 µm o.d) due to the flexibility of the Teflon. (C) Schematic diagram of the coaxial sheath-liquid-assisted electrospray ionisation (ESI) sprayer used. Droplets emerging from the inner capillary are being sheathed by a liquid from the middle capillary and are nebulized by a nebulizing gas (nitrogen) from the outer capillary towards the MS inlet.

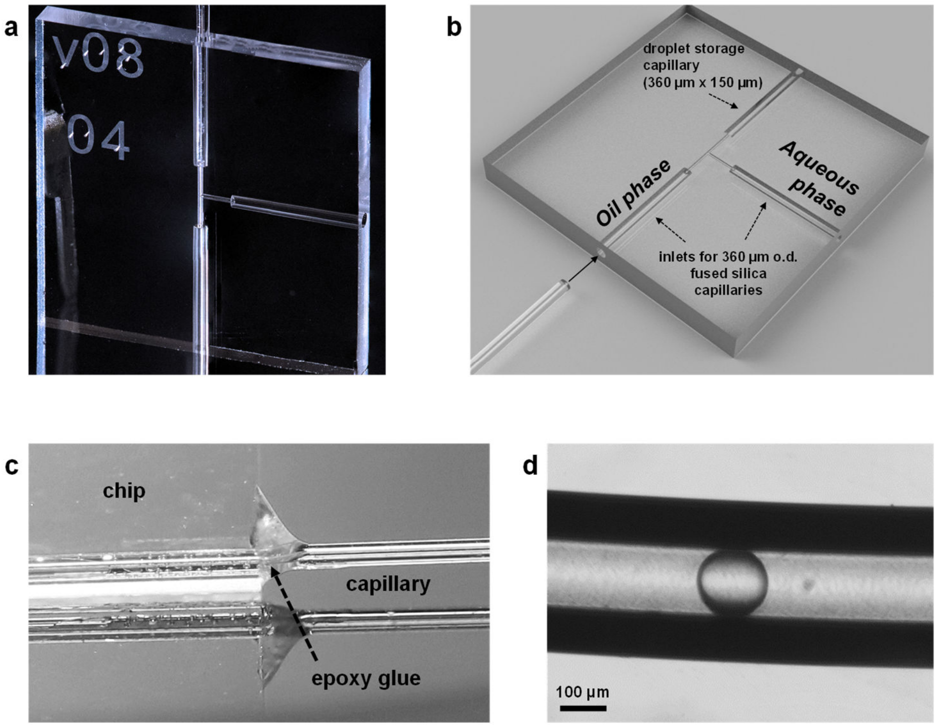


**Fig. S4** Monolithic tee junction chip. (a) Photograph of a chip (the dimensions of the chip are 10 mm x 10 mm x 1 mm). (b) Schematic of the chip with labeling of the capillaries used. (c) Photograph of the sealing of the capillary-chip connection with epoxy glue. (d) Droplet in a PFA tubing.

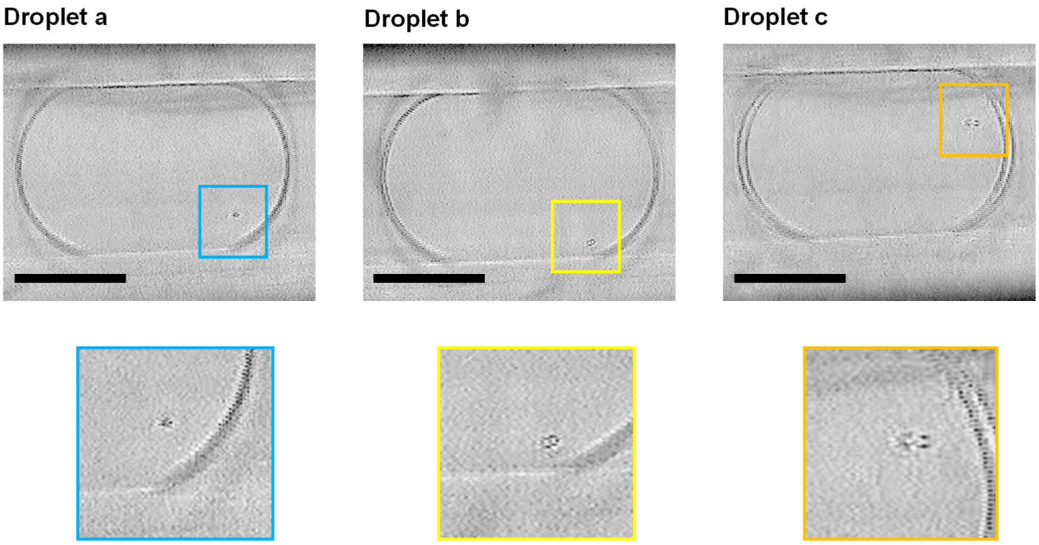


**Fig. S5** Extracted still images of droplets with single cells. Yeast cells were detected as different shapes depending on their position in the droplet during the imaging, e.g. with an emerging bud as in droplet b and c. Scale bar: 100 µm.

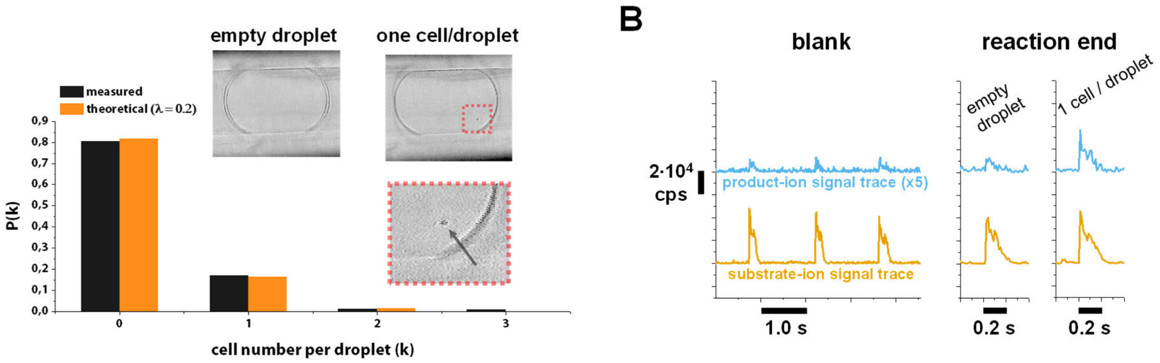


**Fig. S6** Visual and ESI-MS-based droplet analysis at single-cell resolution. (a) Theoretical Poisson distribution $P(k) = \lambda^k e^{-k} (k!)^{-1}$ for λ = 0.2 and the experimental distribution (N=222) of the cell number k per droplet. Additionally, extracted still images of an empty droplet and a droplet containing a single cell are inserted. Here, *P(k)* is the probability to find *k* cells in a droplet with an average cell count per droplet *λ*. (b) Comparison of blank droplets containing the reaction matrix without the product, and empty droplets and droplets with one cell per droplet at the end of a reaction (25 h).

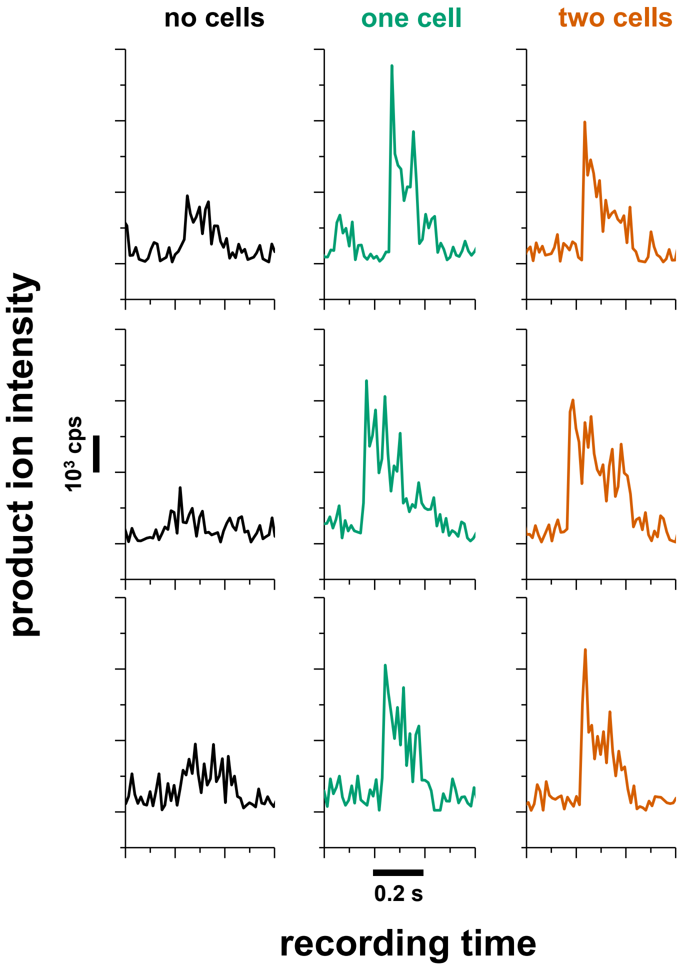


**Fig. S7** Variability of droplet ESI-MS signals. Exemplary product ion signals for droplets with no, one or two cells. The product signal was recorded in the multiple reaction monitoring mode with the m/z ratio pair 133/87.

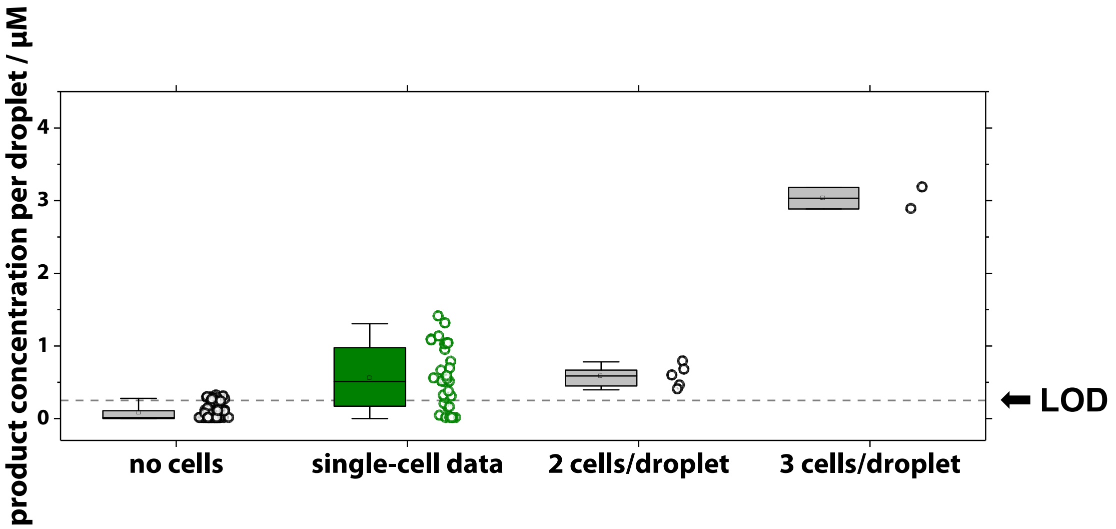


**Fig. S8** Correlation between droplets with different cell amounts to the corresponding product concentration per droplet. Boxplot of the concentration per droplet after 25 h reaction time for droplets with one, two or three cells per droplet ($N_{total}$=136).

**Supplemental Videos**

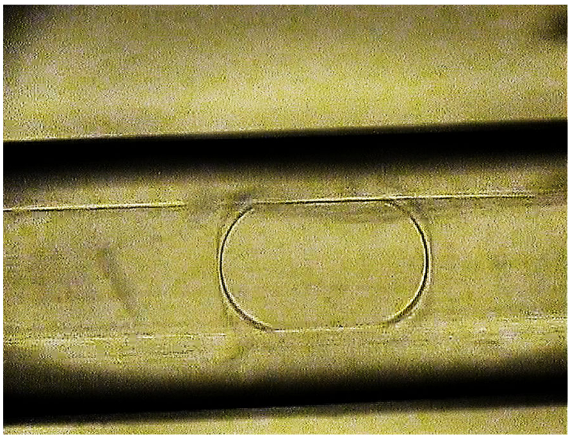

**Video S1** Empty droplets**.** 3-nL-sized droplets are flowing inside a PFA capillary (360 µm o.d. /150 µm i.d.) and are transilluminated from the bottom.

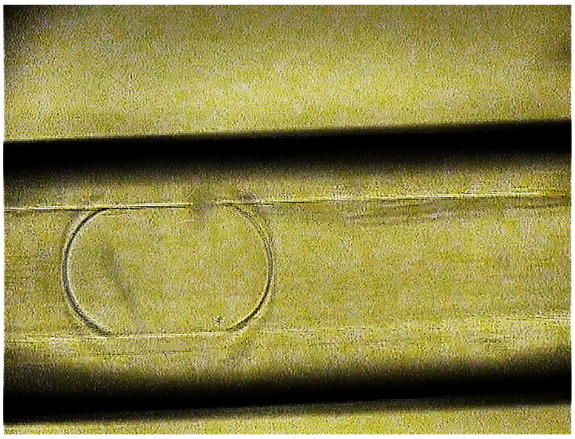

**Video S2** Droplet with one single cell. 3-nL-sized droplet containing one single cell (*S. cerevisiae*) is flowing inside a PFA capillary (360 µm o.d. /150 µm i.d.) and is transilluminated from the bottom.

## References


1. D. A. Treco, F. Winston, Curr. Protoc. Mol. Biol. **2008**, *82*,1-12.
